# In Vitro and In Vivo Antiurolithic Effect of Betulinic Acid Obtained from *Citharexylum mirianthum*

**DOI:** 10.3390/plants13152141

**Published:** 2024-08-01

**Authors:** Luísa Nathália Bolda Mariano, Gabriela Vequi, Rita de Cássia Vilhena da Silva, Anelise Felício Macarini, Anelize Dada, Thaina Mariz Costa, Murilo Morales Omena, Christiane Regina Pamplona Pereira, Valdir Cechinel-Filho, Rivaldo Niero, Priscila de Souza

**Affiliations:** 1 Postgraduate Program in Pharmaceutical Sciences, Núcleo de Investigações Químico-Farmacêuticas (NIQFAR), Universidade do Vale do Itajaí (UNIVALI), Itajaí 88302-901, SC, Brazilritasilva@univali.br (R.d.C.V.d.S.);; 2 School of Health Sciences, Universidade do Vale do Itajaí (UNIVALI), Rua Uruguai 458 Centro, Itajaí 88302-901, SC, Brazil

**Keywords:** urolithiasis, phenolic acids, kidney function, calcium oxalate

## Abstract

The study aimed to investigate the potential antiurolithic effects of extracts, fractions, and betulinic acid (BA) from *Citharexylum mirianthum*. In vitro analysis involved precipitating calcium oxalate (CaOx) crystals in urine. For in vivo studies, rats were divided into four groups: naive; vehicle; potassium citrate (KC); and BA. Urolithiasis was induced using ethylene glycol and ammonium chloride. After seven days, urine, blood, and kidney tissues were evaluated. The results showed that methanolic extract, hexane, dichloromethane, and ethyl acetate fractions, as well as BA, reduced CaOx crystal formation. In vivo, the vehicle-treated group exhibited reduced urinary volume and Na^+^ excretion, while the BA-treated group showed restored urinary volume and Na^+^ excretion similar to the naive group. BA also significantly reduced urinary monohydrate and dihydrate crystal formation, comparable to the KC group. Other urinary parameters remained unchanged, but plasma analysis revealed decreased Na^+^, K^+^, and Ca^2+^ in the KC group. Renal tissue analysis indicated reduced lipid hydroperoxides and increased reduced glutathione in all urolithiasis groups, with unchanged nitrite levels. BA treatment also improved renal corpuscle morphology. Overall, our findings demonstrate that treatment with BA effectively prevented kidney damage induced by EG+AC ingestion, thereby improving renal function in the urolithiasis model.

## 1. Introduction

Nephrolithiasis, a prevalent urological disease, affects both Western and Eastern populations [1], with a higher incidence in men [2,3]. The risk of recurrence remains notably high [4,5]. Factors such as diets rich in sodium, oxalate, animal proteins, and low fluid intake and excess calcium are often associated with stone formation. Another well-known risk factor for nephrolithiasis is obesity, diabetes, inflammatory bowel disease, and hypertension [4]. Still linked to overweight, bariatric surgery has been studied for its link to a higher incidence of nephrolithiasis occurring 1.5 to 3.6 years post-procedure. The complex pathophysiology includes factors such as hyperoxaluria, hypocitraturia, aciduria, and urine supersaturation with calcium and oxalate [6].

The main pathophysiology associated with the genesis of stones is the supersaturation of substances in the urine, such as hypercalciuria, hyperoxaluria, hyperuricosuria and cystinuria. This leads to precipitation and the formation of crystals, which can aggregate and form kidney stones [5]. Around 80% of lithiasis has calcium oxalate (most common) and/or calcium phosphate in its composition [2].

Natural resources have been used by civilizations for thousands of years. There are references of around 1700 medicinal plants reported by the Egyptians, in addition to records found in imperial China, around 3000 BC, as well as Greeks, Assyrians, and others. Such is the importance of herbal medicine, which the World Health Organization recognizes as an important factor in primary health care. Thus, the search to understand active ingredients, mechanism of action, and scientific basis takes an important place in this scenario [7].

Betulinic acid is a pentacyclic triterpene, obtained from the oxidation of betulin, which is very abundant and present in nature [8], and also obtained from *Citharexylum mirianthum* Cham., of the Verbenaceae family [9,10,11]. This compound has been associated with many biological benefits, including antiviral activity, antibacterial, antimalarial, antiallergic, antiangiogenic, anti-inflammatory, antifibrotic, anticonvulsant and hepatoprotective [12]. Studies have indicated that betulin and betulinic acid has a protective action on kidney cells during treatment with cisplatin (a widely used chemotherapy compound). In research carried out in the Republic of Korea, where kidney damage was induced with cisplatin and the protective effect of both compounds was evaluated, it was found that they reduced the damage to 80%, proving its nephroprotective properties [8].

Therefore, this natural bioactive derived from birch, with high antioxidant and anti-inflammatory potential, is widely distributed in plant derivatives used in folk medicine [12]. Recent studies by Pereira et al. [10] have shown that betulinic acid exhibits diuretic and natriuretic effects. This activity is likely associated with the stimulation of cholinergic receptors, and the synthesis of prostaglandins. By promoting urine production, diuretics can help prevent the formation of kidney stones by diluting urine and reducing the concentration of stone-forming substances.

In a pharmacological review carried out by Rastogi et al. [13], betulinic acid was attributed joint protection effects by inhibiting the degradation of collagen and proteoglycans. Thus, among the antirheumatic effects, it was noted that this substance can reduce the concentration of urate and has great potential to inhibit xanthine oxidase, important in the mechanisms that form renal lithiasis.

Based on the potential pharmacological effects of betulinic acid that could benefit in the therapeutic management of disorders associated with kidney stones, this study aimed to investigate the potential in vitro antiurolithic effect of extracts, fractions, and betulinic acid derived from *C. mirianthum*. In addition, the in vivo effect of betulinic acid was accessed in a model of ethylene glycol and ammonium chloride-induced urolithiasis. Various parameters were assessed in the collected urine, blood, and kidney tissue samples at the conclusion of the experiment.

## 2. Results and Discussion

The interest in studying the antiurolithic effect of preparations obtained from *C. mirianthum* and botulin acid arose after the results obtained with betulinic acid, isolated from the hexane fraction (HEX) of *C. mirianthum* in a diuresis model in rats [10]. Administered orally to rats at a dose of 1 mg/kg, it substantially increased urine and sodium output without altering potassium and chloride levels. This effect was blocked by atropine (a cholinergic receptor antagonist) and indomethacin (a cyclooxygenase inhibitor), indicating involvement of these pathways in betulinic acid’s diuretic action. The findings of this study open perspectives for future applications of betulinic acid in which modulation of the renal function is desired.

Betulinic acid is a pentacyclic triterpenoid compound consisting of five rings, including a pentacyclic ring system. The structure features a hydrophobic backbone with hydroxyl (OH) groups attached to the rings. It has a molecular formula of C_30_H_48_O_3_ and a molecular weight of approximately 456.71 g/mol. Due to its biological activities, betulinic acid is studied for potential applications in pharmaceuticals, cosmetics, nutraceuticals, and agrochemicals. These characteristics make betulinic acid a significant compound in medicinal chemistry and biotechnology, with ongoing research exploring its therapeutic and industrial potentials.

**Figure 1:**
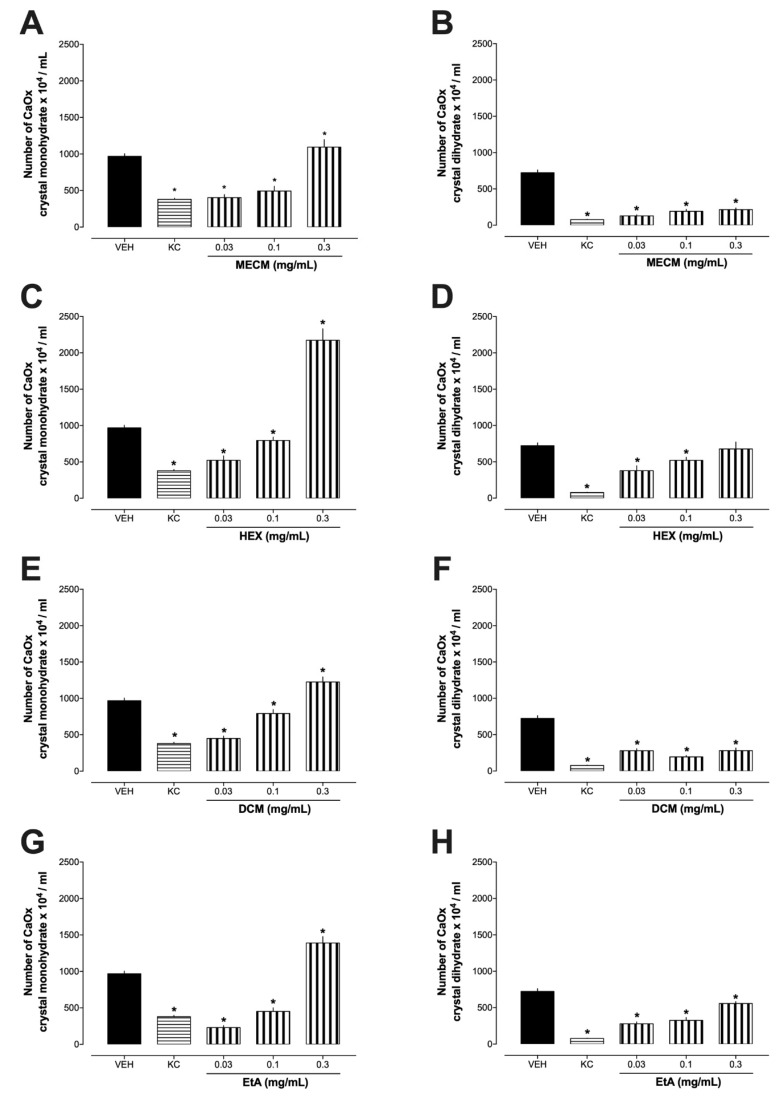
Inhibitory effects of different preparations obtained from *C. mirianthum* on urinary calculus induced by CaOx precipitation. Panel (**A**,**C**,**E**,**G**) CaOx monohydrate crystals. Panel (**B**,**D**,**F**,**H**) CaOx dihydrate crystals. * *p* < 0.05 in comparison the vehicle (VEH) group. CaOx: calcium oxalate. KC: potassium citrate (10 mg/mL). MECM: methanolic extract from *C. mirianthum*. HEX: hexane fraction. DCM: dichloromethane fraction. EtA: ethyl acetate fraction.

Initially, the antiurolithic effect of the methanolic extract of *C. mirianthum* (MECM) and fractions of n-hexane (HEX), dichloromethane (DCM), and ethyl acetate (EtA) were investigated in in vitro urinary stone methodology. As shown in Figure 1, potassium citrate (KC; 10 mg/mL), the positive control group, decreased the total number of CaOx monohydrate and dihydrate crystals when compared to the VEH sample. KC is commonly used in the management and prevention of kidney stones, particularly those composed of calcium oxalate or uric acid. It increases the pH of urine, making it less acidic. This helps prevent the formation of certain types of kidney stones, such as uric acid stones, which tend to form in acidic urine. By increasing urinary citrate levels, KC helps inhibit the formation of calcium oxalate stones. Citrate binds to calcium in the urine, preventing it from forming crystals that can lead to stone development. It is prescribed by healthcare providers based on the specific type of kidney stone and the patient’s urinary pH and citrate levels. Regular monitoring and adjustments may be necessary to optimize treatment effectiveness and safety, once it can also potentially cause adverse effects in some individuals [14,15].

The MECM at concentrations of 0.03 and 0.1 mg/mL reduced the formation of monohydrate and dihydrate forms of CaOx crystals (Figure 1A,B). However, at 0.3 mg/mL concentration, MECM significantly increased the formation of monohydrate crystals compared to the VEH sample. All fractions (Figure 1C,E,G) reduced monohydrate crystal formation at 0.03 and 0.1 mg/mL concentrations but increased it at 0.3 mg/mL Conversely, fractions (Figure 1D,F,H) consistently reduced dihydrate crystal formation across all concentrations. These results are important, since about 92% of the crystals originates from calcium, of which 46% are formed by CaOx [16]. The monohydrate crystal is the primary component formed in cases of urolithiasis or kidney stones [17].

**Figure 2:**
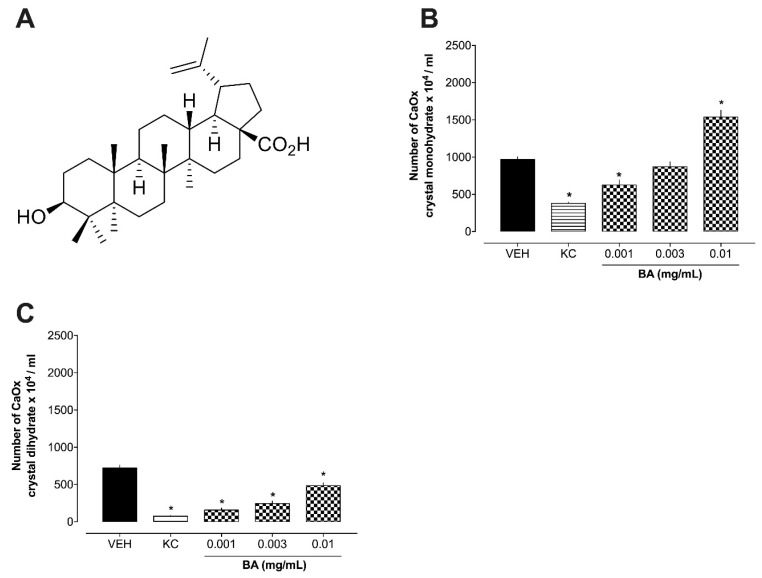
Inhibitory effects of betulinic acid on urinary calculus induced by CaOx precipitation. Panel (**A**) Molecular structure of the betulinic acid (BA). (**B**) CaOx monohydrate crystals. (**C**) CaOx dihydrate crystals. * *p* < 0.05 in comparison the vehicle (VEH) group. CaOx: calcium oxalate. KC: potassium citrate (10 mg/mL). BA: betulinic acid.

Next, we analyzed the effect of betulinic acid (BA), isolated from the hexane fraction, as depicted in Figure 2. As shown (Figure 2C), BA reduced the formation of dihydrate forms of CaOx crystals across all concentrations (0.001, 0.003, and 0.01 mg/mL) and reduced monohydrate forms only at a concentration of 0.001 mg/mL (Figure 2B). However, at a concentration of 0.1 mg/mL BA, there was a substantial growth in the formation of monohydrate forms of CaOx crystals. Aligned with the literature, recent research has shown the antiurolithic effects of plant species or isolated compounds using the CaOx model [18,19,20]. The use of in vitro models can reduce costs and the time required to test the initial biological activity of a plant, before moving on to more complex studies in animal models.

Based on the results described above, both the extract, fractions and isolated compound exhibited variable effects on CaOx crystal formation in vitro. At lower concentrations, all the preparations effectively reduced the formation of monohydrate and dihydrate forms of CaOx crystals, indicating potential antiurolithic properties. In addition to the efficacy demonstrated for species *C. mirianthum*, these initial data suggest that BA appears to be one of the extract’s biological activity markers and promising for future studies.

For that, the effects of BA (1 mg/kg) were explored on in vivo kidney stone methodology, through the administration of ethylene glycol and ammonium chloride. This dosage was selected because it represented the lowest dose that exhibited a diuretic effect in the study conducted by Pereira et al. [10]. Ethylene glycol and ammonium chloride (EG-AC)-induced urolithiasis is a commonly used experimental model to simulate the formation of kidney stones in animals. EG is metabolized in the body to oxalate, which combines with calcium to form calcium oxalate crystals, a predominant component of kidney stones, while AC acidifies urine, promoting the formation of acidic conditions that favor the precipitation of calcium oxalate and other types of crystals.

**Figure 3:**
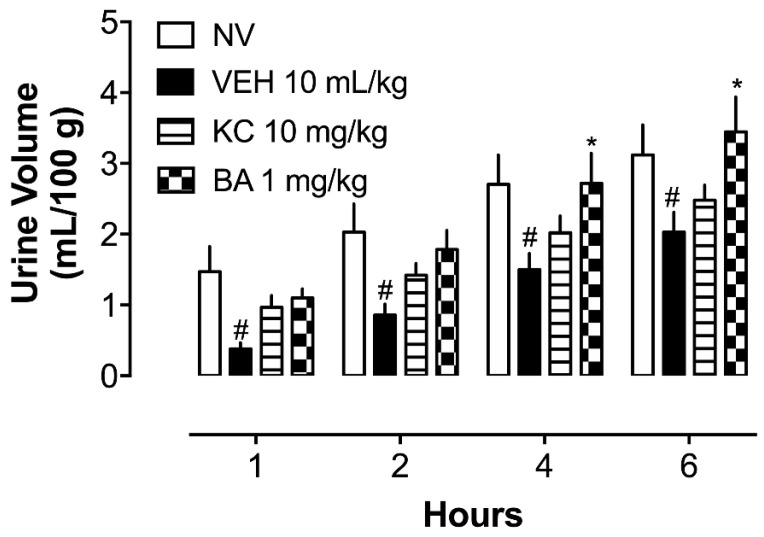
Urine volume of rats after seven days of urolithiasis induction. # *p* < 0.05 in comparison to the NV group. * *p* < 0.05 in comparison to the VEH group. VEH: Vehicle (water plus 1% tween; 10 mL/kg). KC: potassium citrate (10 mg/kg). BA: betulinic acid (1 mg/kg).

As depicted in Figure 3, following seven days of urolithiasis induction, urine volume was monitored over a six-hour period. Analysis of the urine volume revealed a significant reduction in the VEH group compared to the naive group (NV). Conversely, oral administration of BA (1 mg/kg) significantly increased the urine volume compared to the VEH group, effectively restoring renal function to levels comparable to those observed in the NV group.

Additionally, urinary parameters were assessed (Table 1). As denoted, pH and urinary conductivity values did not exhibit significant differences, suggesting that neither the urolithiasis induction model nor the treatments tested induced notable metabolic changes. However, there was a significant decrease in sodium excretion in the urine of the vehicle-treated group compared to the NV group (Table 1). Conversely, oral administration of BA (1 mg/kg) prevented this alteration compared to the vehicle-treated group. No statistically significant differences were observed in urinary concentrations of Cl^−^, K^+^, and Ca^2+^ ions.

Urinary and serum uric acid levels exhibited no significant differences, as illustrated in Table 1 and Table 2, respectively. Assessing these levels is crucial for estimating the potential protection against or risk of calcium phosphate supersaturation in urine. Increased uric acid levels in urine can favor the pH conditions conducive to calcium oxalate crystal formation. Additionally, uric acid is pertinent for distinguishing between monohydrate and dihydrate crystal forms, with monohydrate crystals linked to hyperoxaluria and dihydrate crystals to hypercalciuria [21].

Upon analyzing serum samples (Table 2), significant alterations were observed solely in the KC (10 mg/kg) group, particularly in sodium, potassium, and calcium ion levels. This finding is particularly noteworthy, as it suggests that the KC (10 mg/kg) compound might potentially lead to hyponatremia and hypokalemia due to decreases in serum sodium and potassium levels, respectively. Consequently, this highlights the need for further research to develop alternative medications with fewer adverse effects.

**Figure 4:**
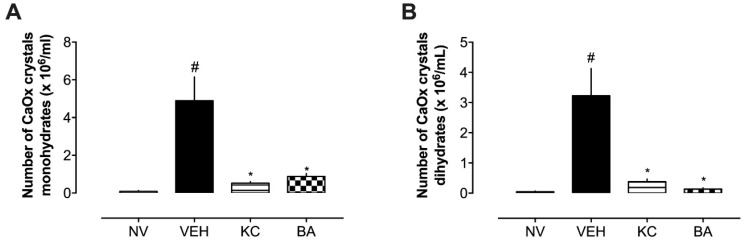
Effect of treatment with BA (1 mg/kg) on the number of calcium oxalate crystals in the urine after seven days of induction of urolithiasis. Panel (**A**) CaOx monohydrate crystals, and (**B**) CaOx dihydrate crystals. * *p* < 0.05, compared to NV group. # *p* < 0.05, compared to VEH group. CaOx: calcium oxalate. VEH: Vehicle (water plus 1% tween; 10 mL/kg). KC: potassium citrate (10 mg/kg). BA: betulinic acid (1 mg/kg).

Using an optical microscope, the urinary stone tests were evaluated in fresh urine. Monohydrate crystals typically appear as dumbbell-shaped or oval crystals under microscopic examination, while the dihydrate crystals often appear as envelope-shaped or bipyramidal crystals under the microscope. Regarding the solubility, monohydrate crystals generally less soluble in urine, making them more likely to contribute to stone formation. On the other hand, dihydrate crystals more soluble in urine compared to monohydrate crystals but can still contribute to stone formation under certain conditions. From the images obtained (Figure 4), it is evident that there was a greater formation of both monohydrate (Figure 4A) and dihydrate crystals (Figure 4B) in the VEH group. This result aligns with previous studies demonstrating that the combination of ammonium chloride and ethylene glycol reliably induces high rates of renal crystal deposition. For this reason, ethylene glycol ingestion in rats has been widely adopted as an experimental model for studying nephrolithiasis [22]. After administration of ethylene glycol (1%) and ammonium chloride (1%) for seven days, associated with treatments with KC (10 mg/kg) and BA (1 mg/kg), it was observed that KC significantly reduced the monohydrate stones present in the urine, and that BA showed a similar result. In relation to dihydrate crystals, both treatments significantly reduced the presence of these crystals (Figure 4B).

**Figure 5:**
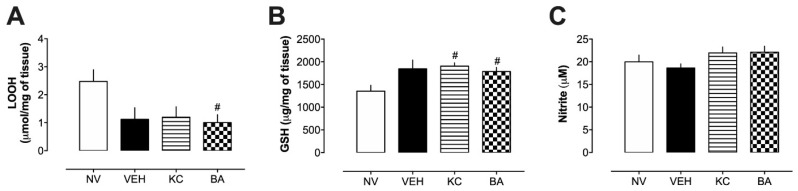
Effect of betulinic acid (BA) on renal oxidative stress markers after 7 days of urolithiasis induction. (**A**) Lipid hydroperoxides (LOOH) content, (**B**) reduced glutathione (GSH) levels, (**C**) nitrite levels in kidney samples collected from rats. # *p* < 0.05 in comparison to the NV group. VEH: Vehicle (water plus 1% tween; 10 mL/kg). KC: potassium citrate (10 mg/kg). BA: betulinic acid (1 mg/kg).

Studies have indicated that calcium oxalate (CaOx) stones can induce tissue damage through the generation of reactive oxygen species [23,24]. Additionally, oxidative stress and reduced nitric oxide (NO) levels are implicated in kidney diseases [25]. Upon analyzing kidney tissue (Figure 5), we observed that all groups subjected to the urolithiasis protocol exhibited decreased levels of lipid hydroperoxide (LOOH) (Figure 5A). Moreover, elevated levels of reduced glutathione (GSH) were observed in the VEH, KC, and BA groups (Figure 5B). Lipid peroxidation is associated with numerous diseases and can be initiated by various oxidants. Its effects can result in cellular dysfunction and tissue damage, serving as an indirect indicator of oxidative stress levels within tissues [26]. Considering the reduction in LOOH content observed in the results, despite the potential presence of increased oxidative stress, it suggests that the antioxidant system may be more actively engaged in repairing and/or preventing oxidative damage. The elevated levels of GSH across all urolithiasis-induced groups support this hypothesis, indicating enhanced antioxidant defense mechanisms. Additionally, nitrite levels were assessed as a marker of nitric oxide (NO) production, given its involvement in various physiological kidney processes, including diuresis and natriuresis [25]. As depicted in Figure 5C, there were no significant changes in nitrite levels following the induction of urolithiasis for seven days.

**Figure 6:**
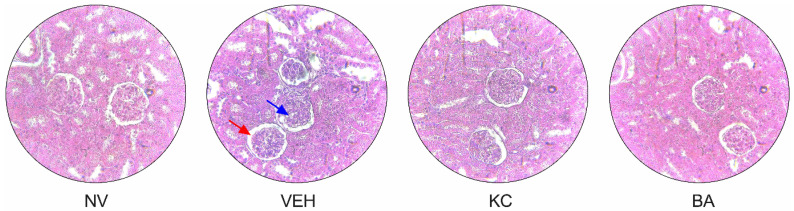
Representative images of renal tissue morphology stained with hematoxylin and eosin (H&E). The red and blue arrows indicate the region of the Bowman’s capsule and the renal glomerulus, respectively. NV: naive. VEH: vehicle (water plus 1% tween; 10 mL/kg). KC: potassium citrate (10 mg/kg). BA: betulinic acid (1 mg/kg).

Finally, Figure 6 illustrates the histological results of the kidneys from animals after seven days of treatment. The VEH group exhibited mesangial space rupture and increased thickening of Bowman’s capsule compared to the NV group. The changes observed in the groups treated with KC and BA were less pronounced compared to those in the VEH-treated group. CaOx crystals can damage renal epithelial cells, leading to the secretion of free radicals [27] and administration of antioxidants can prevent crystal retention [28]. By counteracting free radicals and lowering oxidative stress, antioxidants preserve the integrity of renal epithelial cells and prevent conditions conducive to crystal retention. This protective effect can reduce the likelihood of crystal formation and growth, thereby aiding in the prevention of kidney stones. However, we cannot suggest that BA through antioxidant actions contributed to the beneficial effects observed in this study. The data point to protection and modulation of renal function, and to suggest mechanisms, further studies are necessary.

## 3. Materials and Methods

### 3.1. Obtaining Extract, Fractions, and Betulinic Acid from C. myrianthum

The extraction, fractionation, and isolation of betulinic acid were performed following the method described by Pereira et al. [11]. Briefly, aerial parts of *C. myrianthum* were harvested in June 2017 in Itajaí, SC, Brazil, with a specimen archived at the Herbarium Barbosa Rodrigues (code HBR56933). The leaves were dried, ground, and subjected to maceration in methanol (MeOH) for seven days. The methanol was then evaporated under reduced pressure using a rotary evaporator, resulting in a crude methanolic extract. This extract was dissolved in a MeOH mixture (9:1) and sequentially partitioned with n-hexane (HEX), dichloromethane (DCM), and ethyl acetate (EtA). The hexane fraction was chromatographed on a silica gel column with a hexane–acetone gradient, leading to the isolation of betulinic acid (Figure 2A). The isolated compound was then compared with a commercially available standard sample.

### 3.2. In Vitro Protocol of Urolithiasis

The urine samples were divided into aliquots, and urinary stones were induced through calcium oxalate (CaOx) precipitation by adding 40 µL of 0.1 M sodium oxalate per mL of urine. This was done in the presence of various agents: the vehicle (VEH; only urine), potassium citrate (KC; 10 mg/mL), crude methanolic extract (MECM; at concentrations of 0.03, 0.1, and 0.3 mg/mL), fractions (at 0.03, 0.1, and 0.3 mg/mL), and betulinic acid (BA; at concentrations of 0.001, 0.003, and 0.01 mg/mL). The number of crystals was counted in four fields using a light microscope, with each concentration tested in triplicate. Crystal morphology was classified into monohydrate and dihydrate forms.

### 3.3. Animals

Female Wistar normotensive rats, aged three to four months, were utilized in this study. The animals were housed at a controlled room temperature of 22 ± 2 °C, with a 12-h light/dark cycle, and had unrestricted access to water and food. The rats were supplied by Universidade do Vale do Itajaí (UNIVALI). All methodologies and procedures were approved by the Ethical Committee for the Care and Use of Animals at UNIVALI (authorization no. 025/20) and were conducted in accordance with established ethical standards.

### 3.4. Induction of Kidney Stones by Ethylene Glycol and Ammonium Chloride

The study was performed following the method described by Ahmed et al. [29] and Kumar et al. [30] with some modifications. All the animals were weighed and allocated into four groups, each consisting of six to eight rats. The Naive Group (NV) served as a control and received only water throughout the study. The Vehicle (VEH; 10 mL/kg of saline), Potassium Citrate (KC), and Betulinic Acid (BA) groups were administered 1% ethylene glycol (V/V) and 1% ammonium chloride (P/V) in their drinking water for seven days to induce urolithiasis. The VEH group acted as the disease control and received only water. The KC group, treated with 10 mg/kg potassium citrate, served as the standard control. The BA group was treated with betulinic acid at a dose of 1 mg/kg, acting as the test group. All animals were euthanized at the end of the seven-day period.

### 3.5. Assessment of Diuresis

To assess urine analysis, animals from the various experimental groups, as previously described, were placed in metabolic cages for 6 h immediately after receiving their treatments. Cumulative urine excretion was measured and expressed as mL per 100 g of body weight. The concentrations of electrolytes (Na^+^, K^+^, Cl^−^, and Ca^2+^), uric acid, creatinine, urea, as well as urine pH and conductivity, were determined for each urine sample collected during this period.

The concentrations of Na^+^ and K^+^ were measured using a flame photometer (model BFC-300; Benfer, São Paulo, SP, Brazil). Uric acid, creatinine, urea, Cl^−^, and Ca^2+^ levels were determined using colorimetric tests according to the manufacturer’s instructions (Bioclin, Belo Horizonte, MG, Brazil). Conductivity and pH were measured directly in fresh urine samples using a conductivity meter (model DM-32; Digimed, São Paulo, SP, Brazil) and a pH meter (model DM-22; Digimed, São Paulo, SP, Brazil), respectively.

### 3.6. Microscopic Analysis of Urine

Urine was analyzed for crystalluria by placing 5 µL of urine from each animal onto a slide, covering it with a coverslip, and examining it under a light microscope. The number of calcium oxalate crystals was counted, and their morphology was classified as either monohydrate or dihydrate forms.

### 3.7. Serum Analysis

After urine collection, the rats were sacrificed under anesthesia with xylazine (10 mg/kg) and ketamine (80 mg/kg). Blood samples were collected via the abdominal vena cava. The serum was separated and centrifuged at 10,000× *g* rpm for 10 min. The supernatant was analyzed for electrolyte and uric acid levels.

### 3.8. Kidney Analysis

After removing the kidney from all the experimental groups of animals, the tissue was homogenized in a buffer containing 200 mM potassium phosphate (pH 6.5), in a 1:3 weight/volume ratio. The homogenate was used to measure levels of reduced glutathione (GSH), lipid hydroperoxides (LOOH), and nitrite.

### 3.9. Statistical Analysis

The results were presented as the mean ± standard error of the mean (S.E.M) for six to eight animals per group. Statistical analysis was conducted using one-way or two-way analysis of variance (ANOVA) followed by Dunnett’s multiple comparisons test, performed with GraphPad Prism version 7.00 for Mac (GraphPad Software, La Jolla, CA, USA). A *p*-value of less than 0.05 was considered statistically significant.

## 4. Conclusions

The results of this study demonstrated that *C. myrianthum* and its preparations effectively diminished the formation of CaOx crystals. Additionally, treatment with betulinic acid effectively prevented kidney damage induced by ethylene glycol and ammonium chloride ingestion, thereby improving impaired kidney function in the urolithiasis model. Further research is needed to elucidate the renal protective mechanisms of this compound and its effects on renal hemodynamics.

## Figures and Tables

**Table 1:** Impact of betulinic acid (BA) treatment on urinary parameters in rats with urolithiasis. * *p* < 0.05 in comparison to the respective VEH group. ^#^ *p* < 0.05 in comparison to the NV group. VEH: Vehicle (water plus 1% tween). KC: Potassium citrate. BA: Betulinic acid.

Groups	pH	Conductivity (mS/cm)	Na^+^ (mmol/L)	K^+^ (mmol/L)	Cl^−^ (mmol/L)	Ca^2+^ (mg/dL)	Uric Acid (mg/dL)	Creatinine (mg/dL)	Urea (mg/dL)
NV	6.19 ± 0.24	18.94 ± 1.41	83.95 ± 12.54	24.56 ± 2.33	206.7 ± 8.99	17.50 ± 0.30	6.11 ± 0.07	19.08 ± 1.71	10.52 ± 0.66
VEH 10 mL/kg	5.73 ± 0.15	23.40 ± 1.24	56.26 ± 2.83 ^#^	23.62 ± 1.44	207.0 ± 6.02	14.81 ± 1.90	6.28 ± 0.13	9.50 ± 1.24 ^#^	9.55 ± 0.33
KC 10 mg/kg	5.91 ± 0.09	20.18 ± 1.13	73.50 ± 9.85	26.83 ± 5.34	199.2 ± 8.21	19.01 ± 1.11	6.46 ± 0.29	13.53 ± 0.50	9.94 ± 0.70
BA 1 mg/kg	6.03 ± 0.08	22.05 ± 0.97	73.00 ± 7.02	28.26 ± 1.76	212.1 ± 9.16	18.37 ± 1.28	6.22 ± 0.13	14.06 ± 1.53	7.50 ± 0.55 ^#,^*

**Table 2:** Impact of betulinic acid (BA) treatment on plasma parameters in rats with urolithiasis. * *p* < 0.05 in comparison to the respective VEH group. ^#^ *p* < 0.05 in comparison to the NV group. VEH: Vehicle (water plus 1% tween). KC: Potassium citrate. BA: Betulinic acid.

Groups	Na^+^ (mmol/L)	K^+^ (mmol/L)	Cl^−^ (mmol/L)	Ca^2+^ (mg/dL)	Uric Acid (mg/dL)	Creatinine (mg/dL)	Urea (mg/dL)
NV	138.6 ± 2.20	3.15 ± 0.12	206.60 ± 8.22	4.82 ± 0.55	6.36 ± 0.09	0.87 ± 0.17	17.39 ± 1.22
VEH 10 mL/kg	126.0 ± 7.34	3.10 ± 0.16	216.9 ± 15.31	4.91 ± 0.53	6.60 ± 0.14	0.97 ± 0.15	28.81 ± 1.93 ^#^
KC 10 mg/kg	93.86 ± 8.75 ^#,^*	2.25 ± 0.28 ^#,^*	262.2 ± 12.86 ^#,^*	3.26 ± 0.14 ^#,^*	6.51 ± 0.03	0.97 ± 0.13	30.45 ± 2.69
BA 1 mg/kg	109.5 ± 11.72	2.50 ± 0.21	252.5 ± 9.56	3.74 ± 0.05	6.24 ± 0.12	0.77 ± 0.09	35.49 ± 1.39 ^#^

## Data Availability

The raw data supporting the conclusions of this article will be available from the authors upon request.

## Abbreviations

betulinic acid (BA), calcium oxalate (CaOx), dichloromethane (DCM), ethyl acetate (EtA), hexane (HEX), glutathione (GSH), lipid hydroperoxides (LOOH), methanol (MeTOH), methanolic extract of *C. mirianthum* (MECM), naive (NV), nitric oxide (NO), Potassium Citrate (KC), vehicle (VEH).
